# Retrospective analysis of bladder cancer morphology and depth of invasion under cystoscopy

**DOI:** 10.1186/s12894-022-00958-0

**Published:** 2022-01-31

**Authors:** Hu Chen, Yang Hong, Bai Yu, Li Ruiqian, Li Jun, Wu Hongyi, Wang Ziyong, Jiang Haiyang, Zhang Chongjian, Bi Ying, Wang Qilin

**Affiliations:** Department of Urology, Yunnan Cancer Hospital, The Third Affiliated Hospital of Kunming Medical University, Kunming, 650118 Yunnan China

**Keywords:** Bladder cancer, Invasive depth, Morphology cystoscopy

## Abstract

**Background:**

The pathological diagnosis of bladder cancer workup relies on cystoscopy, however, due to sampling restriction, the depth of local invasion is often understaged.

**Methods:**

A total of 386 patients with bladder urothelial carcinoma underwent follow-up. The data collected included age, sex, tumor size, surgical options, histologic grade, invasive depth, lymph node metastasis, and oncological outcomes, and the patients were divided into coral-like and crumb-like groups. These data were analyzed with the chi-square test, binary logistic regression, Kaplan–Meier analysis, univariable and multivariable logistic regression and Spearman correlation test.

**Results:**

Bladder tumor morphology was moderately correlated with invasion depth (ρ = 0.492, *p* < 0.001; Spearman correlation), which was associated with invasion status (HR = 8.27; 95% CI 4.3–15.79, *p* < 0.001). Tumor morphology was not an independent factor for OS but was associated with PFS. Outer invasion depth was an independent factor that was significantly associated with inferior OS and PFS.

**Conclusions:**

Tumor morphology (coral-like and crumb-like) under cystoscopy was related to the depth of invasion. The outer invasive depth of BC was an independent factor that was significantly associated with inferior OS and PFS.

**Supplementary Information:**

The online version contains supplementary material available at 10.1186/s12894-022-00958-0.

## Introduction

Bladder cancer (BC) is the 10th most common cancer worldwide, and the second most common among urologic cancer, with approximately over 540,000 new cases and 200,000 deaths per year [1].

Tumors isolated to non-muscle-invasive bladder cancer (NMIBC) and muscle-invasive bladder cancer (MIBC) [2]. The accurate local staging of bladder carcinoma is key, as it has significant prognostic implications and determines treatment options. Non-muscle-invasive bladder carcinomas (Ta–T1) are suitable for localized treatment, either TURBT or intravesical chemotherapy, while radical cystectomy with LN dissection, remains the gold standard for muscle-invasive disease (≥ T2) [3, 4].

The traditional diagnostic workup relies on cystoscopy and transurethral resection of bladder tumor (TURBT) to confirm the pathological diagnosis and muscle invasive status [3]; however, there is a discrepancy between preoperative pathological staging by cystoscopy biopsy and the postoperative pathologic staging based on surgery, with an inaccuracy rate of 23–50%, mainly due to sampling error, particularly if there is an absence of the muscle layer in the specimen, which would understage the depth of local invasion [5].

In addition, up to 25% of T1 tumors are eventually muscle invasive on subsequent TURBT, which entirely changes therapeutic management [6, 7].

Magnetic resonance imaging (MRI) can effectively cope with such limitations in local staging, and it is increasingly used for the preoperative, due to its high sensitivity to soft-tissue and ability to assess the depth of bladder wall invasion, with a recent meta-analysis reporting a high diagnostic performance in differentiating NMIBC from MIBC [8, 9].

In order to more accurately distinguish the muscular invasion state of bladder cancer, the VI-RADS score was developed in 2018. Multiparametric MRI (mpMRI), which incorporates morphological T2-weighted imaging (T2WI) alongside the functional sequences of diffusion-weighted imaging (DWI) and dynamic contrast-enhanced (DCE) imaging, has been shown to further improve the accuracy of primary and recurrent tumor detection and local staging [9, 10].

The sensitivity and specificity of a VI-RADS score of 3 or greater were 87.1% (95% CI 78–93%) and 96.5% (95% CI 93–98%), respectively [11].

Besides, 29-MHz high-resolution micro-ultrasound (mUS) technology has been suggested as a potential alternative to MRI for the detection of prostate cancer. Some research report that the measures of diagnostic accuracy indexes demonstrate an overall superiority of the mUS compared to MRI, which instead shows an important tendency in lesion upstaging compared to mUS [12].

Cystoscopy is a simple, low-cost routine preoperative operation. Compared with MRI, it can observe the tumor size, position, shape, and surface nourishing blood vessels more clearly and intuitively under direct vision. At present, there are few literature summaries and analyses of tumors with different morphologies and depths of invasion under cystoscopy. A prospective analysis correlating urologist impression at cystoscopy with final pathology. Low grade papillary tumors were accurately identified at cystoscopy 93% of the time, and when information from urine cytology was added the accuracy increased to 99% [13, 14].

We hope to assess the correlation between the morphology of the tumor and the depth of invasion, combined the morphology under cystoscopy with multiparametric mp-MRI to improve the accuracy of local T staging.

## Materials and methods

Electronic medical records were reviewed to identify men treated for bladder cancer at Yunnan Cancer Hospital in China between December 2014 and May 2021. The study protocol was approved by the Yunnan Cancer Hospital Ethics Committee. Among 491 consecutive bladder cancer patients who were treated with surgery, 386 had sufficient follow-up data.

### Entry criteria

1. Tumors were pathologically diagnosed as urothelial carcinoma;
2. Cystoscopy was completed in our hospital before surgery;
3. The muscle tissue was observed in postoperative pathology.

### Exclusion criteria

1. Distant metastasis (M1);
2. Wide base or erosion or ulcer type or urethral mass of tumor;
3. Blurred vision during cystoscopy;
4. Tumors smaller than 1 cm.

**Fig. 1:**
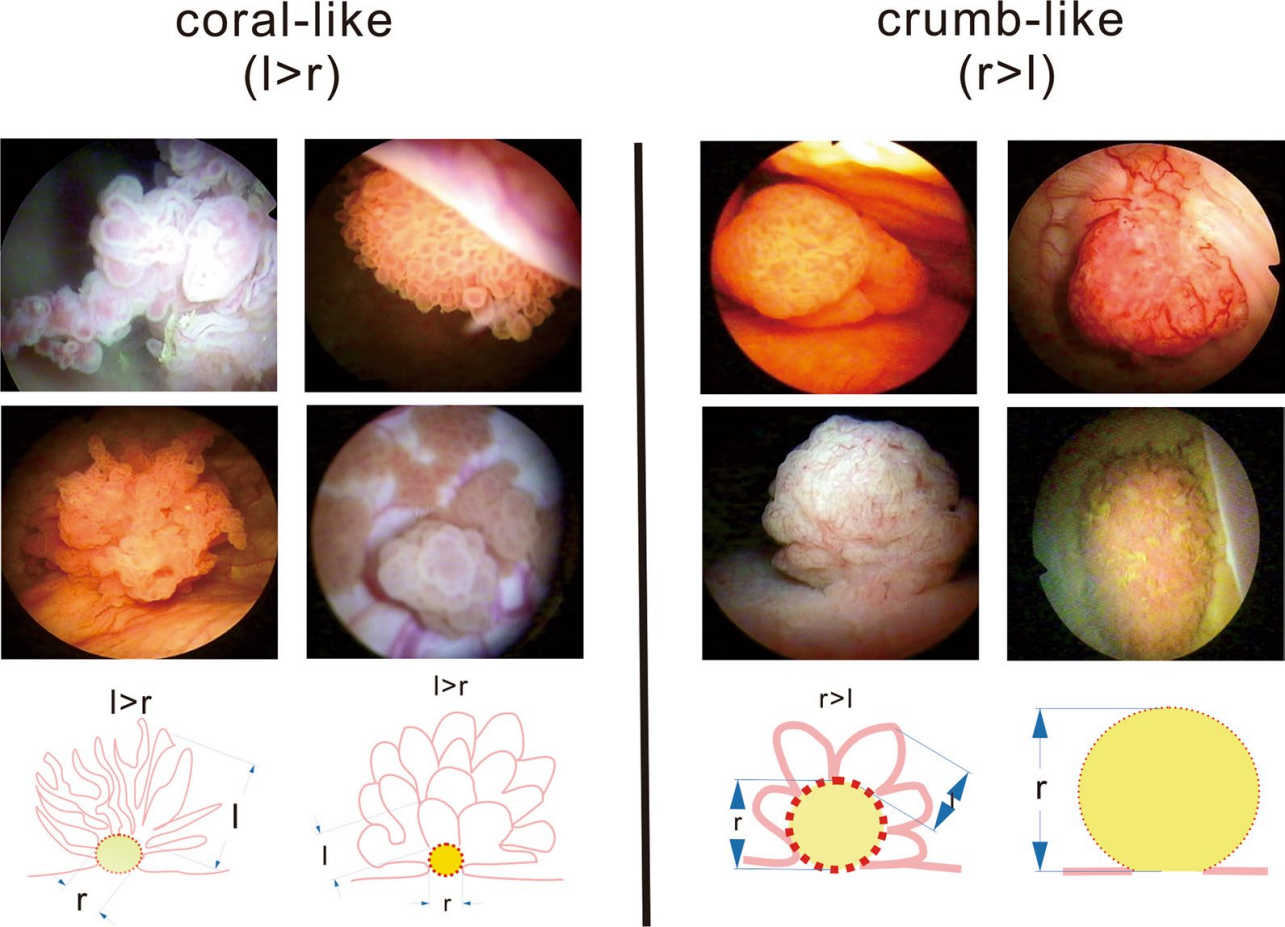
Division into two groups based on the tumor inner circle radius (r) and outer branch length (l) determined by preoperative cystoscopy

According to Fig. 1, the 386 patients were divided into a coral-like group and a clump-like group based on the tumor inner circle radius (r) and outer branch length (l) prior to surgery under cystoscopy. Epidemiological and clinical data, including sex, age, tumor size, surgical options, histologic grade, tumor invasive depth, invasive status, lymph node metastasis status and oncological outcomes (OS and PFS), were collected. Survival analysis was performed by the Kaplan–Meier method for univariable analysis and the Cox regression method for multivariable analysis. The primary endpoints were OS and PFS, which were defined as the times from the date of pathological diagnosis to the dates of death and tumor progression, respectively. The Spearman correlation method was used to evaluate the correlation between the different morphology groups and invasive depths. Univariable (Kaplan–Meier) analysis was performed to determine any differences between the coral-like group and the clump-like group. Multivariable (Cox logistic regression) analysis was used to verify the independence of associations identified in univariable analyses and p-values.

## Results

The mean age (± SD) of the patients was 61.37 ± 11.9 years. The patient data and their relationships to tumor morphology are summarized in Table 1.

**Table 1:** Patients’ characteristics

	Total	Coral-like	Crumb-like	*p* Value
*Sex (n)*	386	264	122	
Male	299(78)	204(77)	95(78)	0.896
Female	87(22)	60(23)	27(22)	
Age (y)	61.37 ± 11.9	60.09 ± 12.57	64.04 ± 10.07	0.526
*Size (n)*				
< 3 cm	271(70)	190(72)	81(66)	0.265
> 3 cm	115(30)	74(28)	41(34)	
*Surgical options (n[%])*				
TURBT	263(68)	198(75)	65(53)	< 0.001
Partial cystectomy	21(5)	11(4)	10(8)	
Radical cystectomy	102(26)	55(20)	47(38)	
*Histologic grade (n[%])*				
PUNLMP	18(5)	17(6)	1(1)	< 0.001
Low grade	105(27)	94(35)	11(9)	
High grade	263(68)	153(58)	110(90)	
*Invasive depth*				
Non-muscle	221(57)	193(73)	28(23)	< 0.001
Lamina propria	87(23)	44(17)	43(35)	
Inner half	33(8)	14(5)	19(16)	
Outer half	16(4)	8(3)	8(6)	
Perivesical	29(8)	5(2)	24(20)	
*Invasive status*				
No	221(57)	193(73)	28(23)	< 0.001
Yes	165(43)	71(27)	94(77)	
*Muscle-invasive status*				
No	307(80)	236(89)	71(58)	< 0.001
Yes	79(20)	28(11)	51(41)	
*Lymph node metastasis*				
No	374(97)	261(98)	113(93)	0.001
Yes	12(3)	3(1)	9(7)	
*Recurrence (n[%])*				
No	350(91)	236(89)	114(93)	0.203
Yes	36(9)	28(11)	8(7)	
*Vital status (n[%])*				
Alive	371(96)	256(97)	115(94)	0.2
Dead	15(4)	8(3)	7(6)	

The chi-square test showed that the coral-like group and clump-like group had significant differences in surgical methods, histologic grade, depth of invasion, and lymph node metastasis.

The Spearman correlation test showed that bladder tumor morphology was moderately correlated with invasion depth (ρ = 0.492, *p* < 0.001) and invasion status (ρ = 0.467, *p* < 0.001), whereas muscle-invasive status (ρ = 0.36, *p* < 0.001), surgical methods (ρ = 0.213, *p* < 0.001), histologic grade (ρ = 0.321, *p* < 0.001), and lymph node metastasis (ρ = 0.167, *p* = 0.001) were weakly correlated, and the remaining variables were not correlated (Table 2).

**Table 2:** Correlation of bladder tumor morphology

Variables	Bladder tumor morphology	*p* Value
	Spearman correlation	
Sex (n[%])	− 0.007	0.897
Age (y)	0.14	0.006
Size (n[%])	0.057	0.267
Surgical type (n[%])	0.213	< 0.001
Histologic grade (n[%])	0.321	< 0.001
Invasive depth	0.492	< 0.001
Invasive status	0.467	< 0.001
Muscle-invasive status	0.36	< 0.001
Lymph node metastasis	0.167	0.001
Recurrence (n[%])	− 0.065	0.204
Vital status (n[%])	0.065	0.202

We used binary logistic regression to analyze the hazard ratios between invasive status and various factors, and the results suggest that tumor morphology was associated with invasive status (HR = 8.27; 95% CI 4.3–15.79, *p* < 0.001), partial cystectomy (HR = 10.65; 95% CI 2.74–41.37, *p* = 0.001) and radical cystectomy (HR = 15.75; 95% CI 7.93–33.98, *p* < 0.001), as shown in Table 3.

**Table 3:** Logistic regression of invasive status and other factors

Variables	Invasive status		
	HR	95% CI	*p*
*Tumor morphology*			
Coral-like	8.27	4.3–15.79	< 0.001
Crumb-like			
*Size*			
< 3 cm	1.30	0.66–2.59	0.447
> 3 cm			
*Surgical type*			
TURBT	Reference	–	–
Partial cystectomy	10.65	2.74–41.37	0.001
Radical cystectomy	15.75	7.93–33.98	< 0.001
*Histologic grade*			
PUNLMP	Reference	–	–
Low grade	0.69	0.08–6.38	0.692
High grade	5.73	0.69–46.96	0.104

Among the 386 patients, 371 survived (median follow-up duration 45 months, interquartile range 29–60 months). In total, 350 patients did not experience recurrence (median follow-up duration 43 months, interquartile range 22–58 months). Disease progression occurred in 36 patients, 20 of whom survived.

**Fig. 2:**
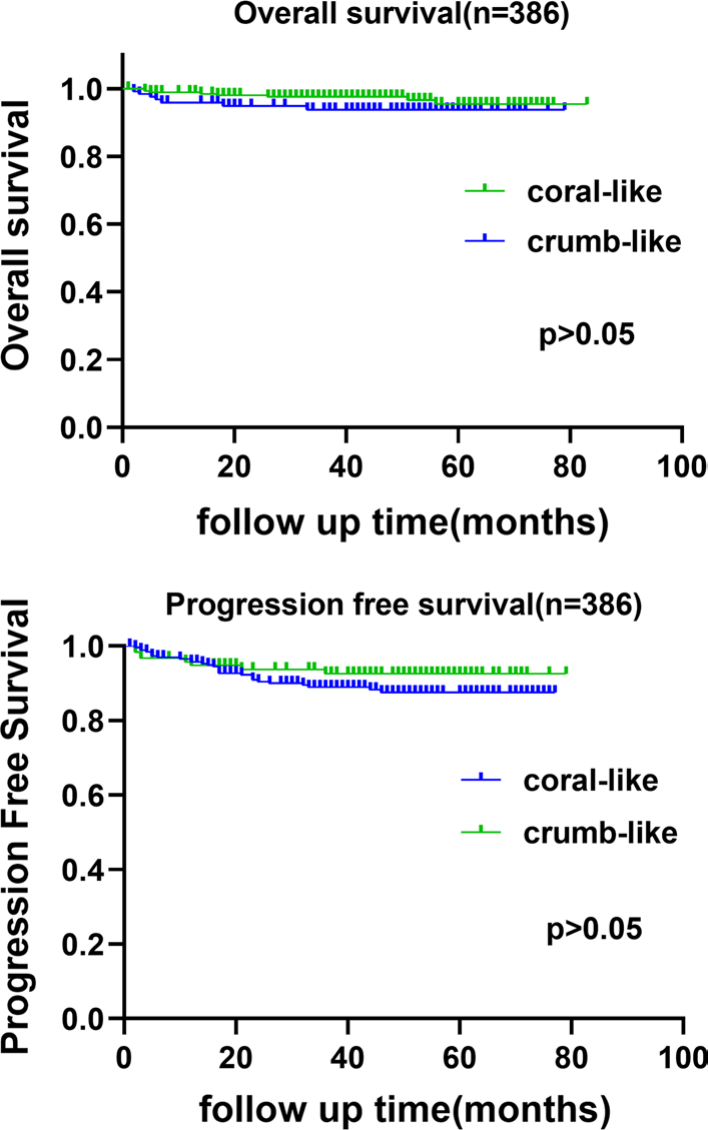
Follow up of OS and PFS

Kaplan–Meier analysis produced the following results: different bladder tumor morphologies (coral-like and clump-like) were not associated with OS, and tumor morphology was not associated with OS (log-rank *p* = 0.206) or PFS (log-rank *p* = 0.250), as shown in Fig. 2.

In univariable analyses, tumor morphology was not associated with OS (HR = 1.9; 95% CI 0.69–5.26, *p* < 0.214). However, coral-like morphology was associated with better PFS (HR = 0.63; 95% CI 029–1.39, *p* = 0.225). In terms of the surgical type, radical cystectomy was significantly associated with inferior OS (HR = 16.49; 95% CI 3.69–73.69, *p* < 0.001) compared to TURBT and not associated with PFS (HR = 1.48; 95% CI 0.73–2.99, *p* = 0.274). Outer invasive depth was significantly associated with both inferior OS (HR = 78.49; 95% CI 9.16–672.83, *p* < 0.001) and PFS (HR = 4.57; 95% CI 1.7–12.33, *p* < 0.002). Perivesical invasive depth was associated with inferior OS (HR = 34.27; 95% CI 3.83–306.93, *p* = 0.002) but was not associated with PFS (HR = 1.93; 95% CI 0.65–5.7, *p* < 0.235). Lymph node metastasis status was significantly associated with inferior OS (HR = 13.46; 95% CI 4.27–42.45, *p* < 0.001) and PFS (HR = 5.25; 95% CI 1.86–14.88, *p* = 0.005).

We performed multivariate analysis, which revealed that tumor morphology was not a significant independent factor for OS (*p* = 0.337) but was associated with PFS (*p* = 0.026). Outer invasive depth was an independent factor that was significantly associated with both inferior OS (HR = 53.74; 95% CI 3.24–892.21, *p* = 0.005) and PFS (HR = 7.73; 95% CI 2.06–29.06, *p* < 0.002). In the multivariate analysis, lymph node metastasis status remained significant for OS (HR = 6.36; 95% CI 1.33–30.44, *p* = 0.021) and PFS (HR = 8.07; 95% CI 1.9–34.27, *p* = 0.005), as indicated in the NCCN and EAU guidelines. However, age, sex, tumor size, surgical type and histologic grade were not significant for OS and PFS in the multivariable analysis (Table 4).

**Table 4:** Cox regression model for OS and PFS

Variables	0S						PFS					
	Univariable			Multivariable			Univariable			Multivariable		
	HR	95% CI	*p*	HR	95% CI	*p*	HR	95% CI	*p*	HR	95% CI	*p*
*Tumor morphology*												
Coral-like	1.9	0.69–5.26	0.214	0.56	0.17–1.84	0.337	0.63	0.29–1.39	0.255	0.34	0.13–0.88	0.026
Crumb-like												
*Size*												
< 3 cm	2.63	0.95–7.25	0.062	0.68	0.23–2.04	0.493	1.29	0.65–2.55	0.463	0.84	0.38–1.84	0.658
> 3 cm												
*Surgical type*												
TURBT	Reference	–	–	Reference	–	–	Reference	–	–	Reference	–	–
Partial cystectomy	6.44	0.58–71.07	0.128	1.22	0.81–18.28	0.887	1.14	0.27–4.86	0.858	0.56	0.11–2.95	0.496
Radical cystectomy	16.49	3.69–73.69	< 0.001	3.05	0.47–19.97	0.250	1.48	0.73–2.99	0.274	0.61	0.21–1.79	0.365
*Histologic grade*												
Punlmp	0.00	–	–	0.00	–	–	0.59	0.08–4.33	0.603	0.56	0.07–4.39	0.584
Low grade	0.176	0.02–1.34	0.093	1.19	0.1–14.0	0.893	0.64	0.28–1.46	0.286	0.63	0.25–1.61	0.337
High grade	Reference	–	–	Reference	–	–	Reference	–	–	Reference	–	–
*Invasive depth*												
Non-muscle	Reference	–	–	Reference	–	–	Reference	–	–	Reference	–	–
Lamina propria	5.30	0.48–58.42	0.713	3.42	0.19–59.73	0.399	0.74	0.27–1.98	0.544	0.87	0.27–2.79	0.811
Inner half	20.12	2.09–193.5	0.009	15.25	0.85–274.96	0.065	1.43	0.48–4.22	0.521	2.65	0.66–10.69	0.170
Outer half	78.49	9.16–672.83	< 0.001	53.74	3.24–892.21	0.005	4.57	1.7–12.33	0.003	7.73	2.06–29.06	0.002
Perivesical	34.27	3.83–306.93	0.002	10.63	0.48–234.39	0.134	1.93	0.65–5.7	0.235	2.14	0.38–12.05	0.389
*Lymph nodes metastic*												
No	13.6	4.27–42.45	< 0.001	6.36	1.33–30.44	0.021	5.25	1.86–14.88	0.002	8.07	1.9–34.27	0.005
Yes												

## Discussion

The diagnosis of bladder cancer requires cystoscopy to obtain the tumor tissue for pathological diagnosis as the gold standard. The pathological results can provide clear information to distinguish between benign and malignant disease and different cancer types. However, it is difficult to obtain samples from the base of the tumor, resulting in inconsistent invasive depth of the preoperative and postoperative pathological diagnoses. The depth of infiltration of the preoperative bladder tissue is often understaged [5].

Preoperatively judging whether the tumor is noninvasive and invades the lamina propria can help the surgeon adjust the depth and breadth of the resection during the operation, maximize the resection to avoid recurrence of the tumor, and improve the prognosis. Second, whether there is muscle invasion directly determines the surgical type as TURBT or radical cystectomy. At present, judging whether there is muscle infiltration is usually determined by MRI. If MRI can be combined with the morphology of the tumor under cystoscopy to improve the accuracy of determining muscle infiltration status, a more reasonable surgical strategy can be chosen to benefit patients.

The relationship between tumor morphology and infiltration was analyzed through Cox regression. The risk of coral-like tumor invasion was 8.27 times that of crumb-like tumor invasion. Therefore, tumor morphology has important value for determining whether infiltration is present, and this information helps in the preoperative staging of tumors and in the selection of surgical type to benefit patients.

Univariate Cox regression indicated that morphology was mainly related to infiltration and had a relationship with the prognostic factors for OS and PFS. The results of multivariate analysis indicated that patients with crumb-like tumors had a longer PFS, which does not match our hypothesis. This may be because there are fewer TURBT options for patients with crumb-like tumors, radical resection or partial resection are other options, and crumb-like tumors are generally subjectively cut deeper during resection. These factors may be related to longer PFS. Second, infiltration reaching the deep muscle layer is a threshold and is associated with worse OS and PFS.

In the next step, prospective experiments will be carried out to compare the accuracy, sensitivity, specificity and positive/negative predictive values separately of preoperative cystoscopy with MRI and mUS on the depth of infiltration. Besides, MRI and mUS will combine to establish a predictive model for preoperative local staging of bladder cancer, hoping to improve the accuracy of judging the status and depth of invasion, which will help in the selection of surgical procedures and the prognosis of patients.

## Conclusions

Tumor morphology (coral-like and crumb-like) under cystoscopy was related to the depth of invasion. The outer invasive depth of BC was an independent factor that was significantly associated with inferior OS and PFS.

## Supplementary Information

**Additional file 1.** Coral-like morphology of bladder cancer under cystoscope.

**Additional file 2.** Crumb-like morphology of bladder cancer under cystoscope.

## Data Availability

The datasets used and analysed during the current study available from the corresponding author on reasonable request.

## Abbreviations

BC: Bladder cancer
TURBT: Transurethral resection of a bladder tumor
OS: Overall survival
PFS: Progression free survival
