# Supplementary figures and images for: Retrospective analysis of bladder cancer morphology and depth of invasion under cystoscopy
Source: BMC Urol. 2022 Jan 31;22:12. doi: 10.1186/s12894-022-00958-0 (PMC8802509; doi:10.1186/s12894-022-00958-0)

# Legend 1 Coral-like morphology of bladder cancer under cystoscope

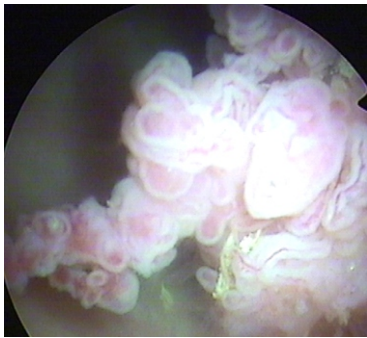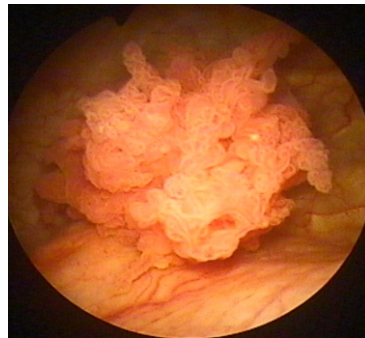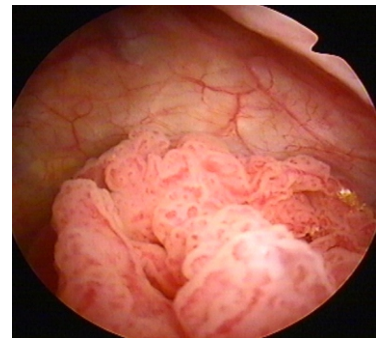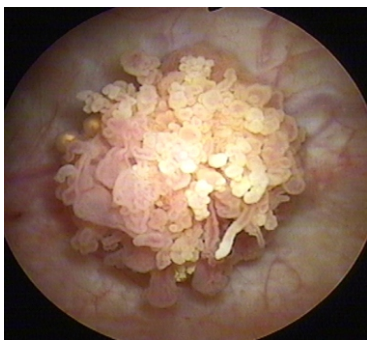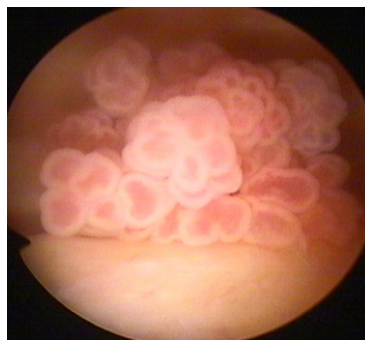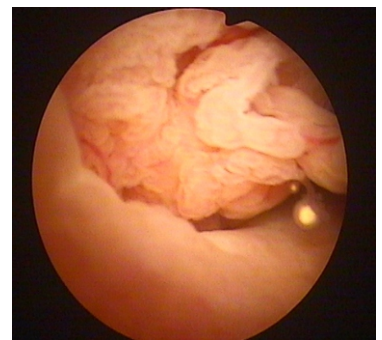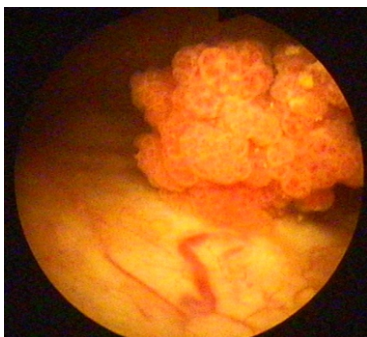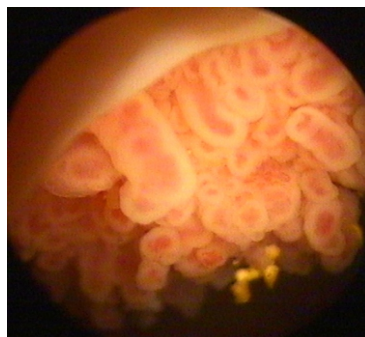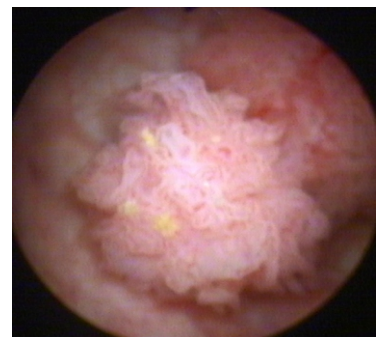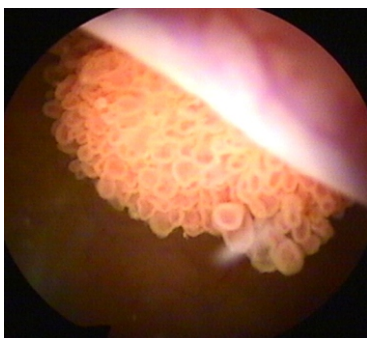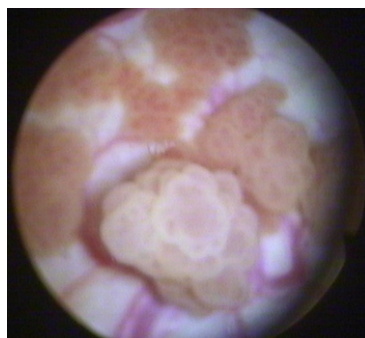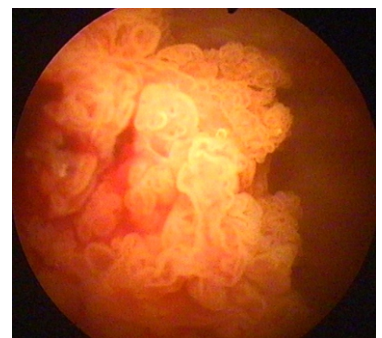

Supplement: Supplementary file 1 — Additional file 1. Coral-like morphology of bladder cancer under cystoscope. [file 12894_2022_958_MOESM1_ESM.pdf]

## Legend 2 Crumb-like morphology of bladder cancer under cystoscope

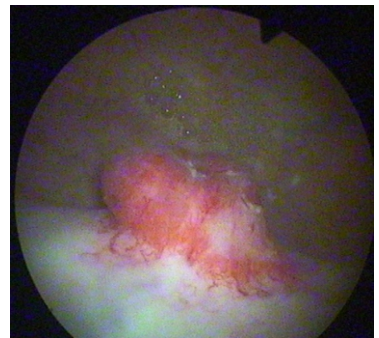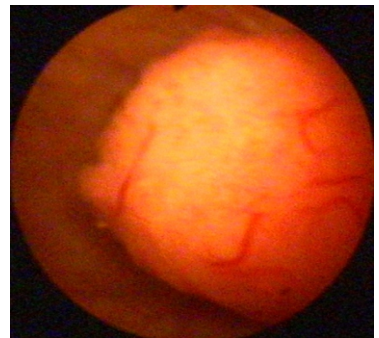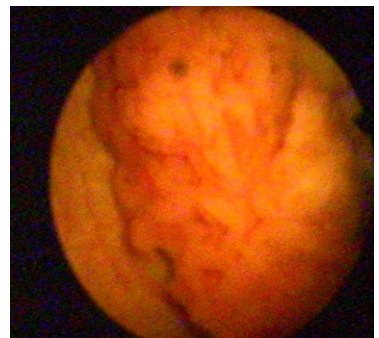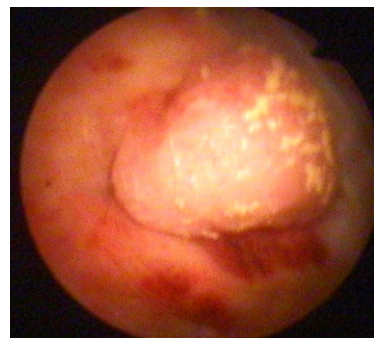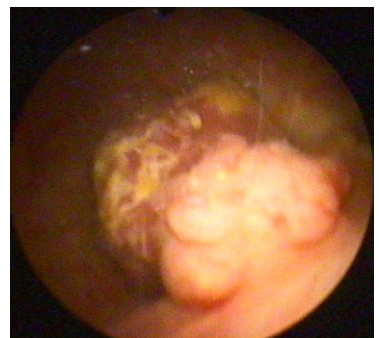

Supplement: Supplementary file 2 — Additional file 2. Crumb-like morphology of bladder cancer under cystoscope. [file 12894_2022_958_MOESM2_ESM.pdf]
